# A Randomized, Multicenter, Double-Blind, Parallel, Non-Inferiority Clinical Study to Compare the Efficacy and Safety of Unicenta and Melsmon for Menopausal Symptom Improvement

**DOI:** 10.3390/medicina59081382

**Published:** 2023-07-28

**Authors:** Seongmin Kim, Sanghoon Lee, Ki-Hoon Ahn, Hyun-Tae Park, Jae-Yun Song, Soon-Cheol Hong, Tak Kim

**Affiliations:** 1 Gynecologic Cancer Center, CHA Ilsan Medical Center, CHA University College of Medicine, 1205 Jungang-ro, Ilsandong-gu, Goyang-si 10414, Republic of Korea; naiad515@gmail.com; 2 Department of Obstetrics and Gynecology, Korea University College of Medicine, 73 Inchon-ro, Seongbuk-gu, Seoul 02841, Republic of Korea

**Keywords:** Kupperman index, menopause, placental extract

## Abstract

This study was conducted to evaluate the efficacy and safety of Unicenta in female subjects with menopausal symptoms by analyzing the changes in the Kupperman index (primary endpoint) and hormonal changes (secondary endpoint). It was a randomized, multi-center, double-blind, parallel, non-inferiority clinical study conducted at two different tertiary medical centers. A Unicenta injection was shown to be non-inferior to Melsmon based on the Kupperman index in both the intent-to-treat and per-protocol populations (*p* = 0.789 and *p* = 0.826, respectively). Additionally, there were no statistically significant differences in hormone levels (estradiol, follicular-stimulating hormone) or in the evaluation of facial flushes. There was no statistically significant difference in the incidence rate of adverse events between the two groups (*p* = 0.505). The study demonstrated that Unicenta is not inferior to Melsmon in terms of the change in the Kupperman index after 12 days of injection. The efficacy and safety of Unicenta were shown, resulting in the improvement of menopausal symptoms.

## 1. Introduction

Historically, the placenta has been used for health and beauty purposes through the ages [1]. It is said that the Chinese emperor Qin Shi Huang used the placenta as one of the elixirs of immortality. There are records of Cleopatra, Yang Guifei, and Marie Antoinette also using the placenta for cosmetic purposes. Various Chinese medical texts such as “Bencao Shuyu” and “Bencao Kangmu” contain records about the placenta. In Korea’s “Donguibogam”, the placenta is mentioned under the name “Jahageo”. In Japan, it is said that the placenta was used in one of the three great secret medicines of the Edo period called “Honyondan” [2]. In modern medicine, the placenta was first used for disease treatment by Dr. Filatov in the 1930s in the Soviet Union [3].

The placenta contains numerous nutrients and functional components as it serves as a substitute for all the necessary functions for the normal development of the fetus [4]. According to current knowledge, the placenta is rich in various amino acids and active peptides, a wide range of vitamins and minerals, enzymes, carbohydrates, nucleic acids, and more [3]. Additionally, the presence of various growth factors, such as nerve growth and hepatocyte growth factors, has been confirmed. Research has shown that placental preparations increase blood circulation in the body, facilitating smooth nutrient supply and promoting the elimination of waste, thus leading to fatigue recovery and strengthening of the body. Studies have also been conducted on various effects, including antioxidant, anti-inflammatory, and skin whitening effects, based on research results from Japan and other countries.

In January 2006, the Korean Ministry of Food and Drug Safety announced the clinical re-evaluation for the safety and efficacy of pharmaceutical products derived from the placenta. Subsequently, after the completion of the re-evaluation, currently in South Korea, there are pharmaceutical products available for sale that include injectables for menopausal symptoms, injectables for improving liver function in chronic liver disease, and oral liquids for fatigue recovery and strengthening the body. In the clinical re-evaluation, the safety and efficacy of the placenta extract injection, Melsmon, were established. Unicenta is also used for the purpose of improving menopausal symptoms, with placental extract as its main ingredient.

In this study, we aimed to evaluate the efficacy and safety of Unicenta in female subjects with menopausal symptoms. By demonstrating the non-inferiority of Unicenta compared to Melsmon, we sought to assess the effectiveness and safety of Unicenta.

## 2. Materials and Methods

### 2.1. Study Design and Population

**Figure 1:**
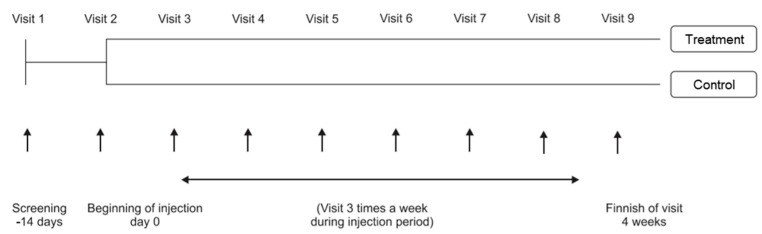
Visit number and follow-up duration of the participants.

This clinical trial is a multicenter, randomized, double-blind, parallel-group, and comparative study designed to evaluate the effects and safety of Unicenta in comparison to Melsmon injections for improving symptoms of menopausal disorders. The visit frequency and follow-up period were as shown in Figure 1.

The screening of participants began on 22 April 2013, and the last participant completed the final visit on 29 January 2014. The inclusion criteria for the study participants were women aged 40 or above who had experienced spontaneous amenorrhea for 12 months or more or had a follicle-stimulating hormone (FSH) level exceeding 40 mlU/mL while experiencing spontaneous amenorrhea for 6 months or more. Additionally, women who had undergone bilateral oophorectomy with a minimum of 6 weeks since the procedure or hysterectomy with a minimum of 6 weeks since the procedure and an FSH level exceeding 40 mlU/mL, were also eligible. Participants were selected based on the severity of facial flushing symptoms documented in the participant’s diary, with a minimum of 3–4 episodes of moderate to severe flushing per day or 20 or more flushing episodes per week. Those with a Kupperman index score of 15 or higher, serum estradiol (E2) levels of 30 pg/mL or lower, the ability to effectively communicate with the investigator, willingness to comply with the trial requirements, and voluntary written consent to participate in the study after receiving a clear explanation of the purpose, methods, and effects of the clinical trial were included.

The exclusion criteria for this study included individuals with a history of hypersensitivity or allergy to the investigational drug or other animal-derived pharmaceuticals; those with concomitant severe menopausal disorders, malignant tumor patients, or with a history of malignancy; uncontrolled hypertension (systolic blood pressure ≥ 170 mmHg or diastolic blood pressure ≥ 110 mmHg); individuals with complications such as uncontrolled diabetes, congestive heart failure, renal failure, pancreatitis, acute or chronic hepatitis/cirrhosis, severe hyperlipidemia, neurological or psychiatric disorders, and systemic infectious diseases including tuberculosis; patients who had undergone hysterectomy with or without bilateral oophorectomy within 6 weeks; individuals with abnormal liver or kidney function indicated by elevated levels of serum glutamic oxaloacetic transaminase (SGOT), serum glutamic pyruvic transaminase (SGPT), bilirubin, or serum creatinine exceeding twice the upper limit of normal; those who had received hormone preparations or analogs other than estrogen and progesterone preparations within the past month; individuals who had participated in another clinical trial within the past 3 months; individuals who were predicted to use prohibited concomitant medications during the trial period; those with a history of alcohol or drug abuse; women aged 40 or above who had suspicious findings of breast malignancy based on breast imaging conducted within 9 months of study registration or had a personal history of breast cancer or a first-degree relative with breast cancer or had an endometrial thickness of 5 mm or more on ultrasound examination and were diagnosed with endometrial hyperplasia or endometrial cancer or had suspicious findings; individuals diagnosed with cervical or uterine cancer or who had suspicious findings based on cervical cytology (Pap smear); patients with endometriosis; individuals receiving inducers of hepatic microsomal enzymes, such as barbital, hydantoins, carbamazepine, meprobamate, phenylbutazone, and rifampicin; patients with jaundice, Dubin–Johnson syndrome, or Rotor syndrome; patients with unexplained genital bleeding; individuals with sideroblastic anemia, porphyria, or other severe metabolic disorders; patients with thrombophlebitis or thromboembolism or a history thereof; patients with cerebrovascular or coronary artery disease, thyroid disorders, acute infectious diseases; previous use of placental preparations; and any other cases deemed unsuitable for participation in the study by the investigator.

All test results conducted until the study was discontinued were used for analysis. Even for prematurely withdrawn participants, efforts were made to conduct the tests during their final visit. Participants who dropped out or had the study discontinued were no longer supported for study-related tests, medical expenses, etc. However, they continued to receive medical care and treatment as patients, and their participation or non-participation in the study did not affect their future medical schedule or content. Furthermore, in cases where adverse reactions occurred, follow-up observations were conducted until the cause of the adverse reaction was identified. The results were reported based on the evaluation criteria, assessment methods, and reporting procedures for safety evaluation, including adverse reactions.

### 2.2. Preparation of Medical Intervention

Unicenta was used as the investigational drug, while Melsmon was used as the comparator in the clinical trial. The assigned study number was sequentially given to participants, and the assigned number of investigational drugs matching the study number was delivered to the administering nurse by the pharmacist. The nurse administered one ampoule (2 mL) per session, three times a week for a total of six doses, with subcutaneous injections performed in subcutaneous tissues, such as the abdomen, upper arm, or thigh. The investigational drug was packaged in six ampoules per package, and the allocation number and visit number were written on each ampoule to minimize errors in administration and delivery.

To prevent biases that could occur when assigning registered participants to either the study group or the control group and to enhance comparability between the two groups, randomization was planned. Stratified block randomization was employed to ensure a balanced distribution between the groups by stratifying participating trial institutions and randomly assigning participants to one of the study groups using blocks.

The participants who were taking medications before 4 weeks prior to their participation in this clinical trial and those medications that were deemed not to have an impact on the interpretation of the trial results, as well as other medications used for the treatment of concurrent conditions or adverse reactions, were administered as concomitant medications after the participant’s judgment and consultation with the sponsor. After the concomitant medication or therapy, the administered medications were reviewed for the medication’s name (product name), daily dosage, reason for administration, duration of administration, and route of administration.

### 2.3. Statistical Analysis

The analysis population of this clinical trial is divided into the intent-to-treat (ITT) analysis population, per-protocol (PP) analysis population, and safety analysis population. The efficacy evaluation was performed for both the ITT and PP analysis populations, but the final assessment for the primary efficacy evaluation variable was based on the PP analysis population. The ITT analysis population was also included for comparison, and individual participant results were provided if there were significant differences. The analysis of secondary efficacy evaluation variables was conducted using both the ITT and PP analysis populations, but the final assessment of the effect was based on the results of the ITT analysis population. The safety analysis population was defined as all participants who received the investigational drug at least once after randomization.

Baseline observational values, including demographic data, were analyzed for this clinical trial. For continuous variables, mean, standard deviation, minimum value, maximum value, etc., were calculated. For categorical variables, absolute and relative frequencies, as well as corresponding percentages, were computed. To compare the trial group and control group regarding demographic variables, Student’s *t*-test was used for continuous variables, while the chi-square test was employed for categorical variables.

The primary endpoint aimed to demonstrate that the change in the Kupperman index from baseline to the final evaluation at 12 days after treatment in the Unicenta group was not inferior to the Melsmon group (control group). To confirm non-inferiority, a one-sided 97.5% confidence interval for the difference in the mean change in the Kupperman index between the Unicenta group and the Melsmon group was calculated. Specifically, if the lower bound of the one-sided 97.5% confidence interval for the difference in the mean change in the Kupperman index was greater than −3.2, it was determined that the Unicenta group was not inferior to the control group. For the secondary endpoints, the mean, standard deviation, and 95% confidence interval were provided separately for the trial group and control group. A Student’s *t*-test was conducted for the comparison between the two groups, while a paired *t*-test was used for within-group changes. To assess the validity, a covariance analysis was performed by including the stratification variables used in random allocation, such as the study institution and trial group status, as covariates, in addition to confounding variables from the baseline demographic data. The analysis results were compared with the overall analysis results for any discrepancies.

The number and percentage of subjects experiencing one or more adverse events were reported for each group, considering the severity of adverse events, their relationship with the investigational drug, and the actions taken and their outcomes. The number and percentage of subjects were described separately for each investigational drug. To compare the incidence rates of adverse events between the two groups, a chi-square test was used. The normal range for clinical trial laboratory tests was based on the respective institution’s reference standards. For safety evaluation results, descriptive statistics (mean, standard deviation, median, minimum value, maximum value) were provided for continuous data. A Student’s *t*-test was conducted for the comparison between the two groups at the screening visit. The paired t-test was used to analyze the differences between before (Visit 1) and after (Visit 8) the application of the investigational drug. The frequency and percentage of subjects who deviated from the normal range before and after the application of the investigational drug were also calculated.

For categorical data among laboratory tests, a chi-square test or Fisher’s exact test, was used to compare differences between groups. McNemar’s test was employed to analyze within-group categorical data before and after the application of the investigational drug. Descriptive statistics (mean, standard deviation, median, maximum value, minimum value, etc.) were provided for continuous variables among clinical examination results. The variables were categorized as below the normal range, within the normal range, or above the normal range based on each institution’s normal range criteria. Categorical variables were classified as normal or abnormal.

## 3. Results

### 3.1. Characteristics of Study Population

**Figure 2:**
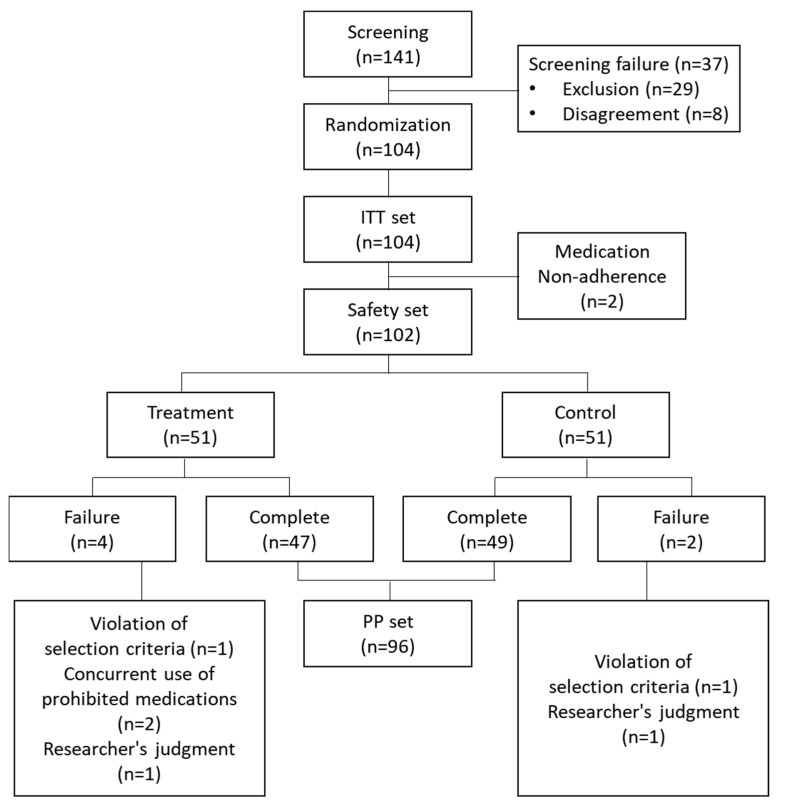
Trial profile. The distribution of participants, along with the number of participants, is presented. ITT, intent-to-treat; PP, per-protocol.

After randomization, the participants were classified into the safety analysis group, ITT analysis group, and PP analysis group based on the application of the investigational drug and the primary efficacy evaluation. The distribution of participants, along with the number of participants, is presented in Figure 2. A total of 141 participants underwent screening, and 104 participants were registered for the application of the investigational drug. Thus, a total of 104 participants were included in the ITT analysis group, and among them, 96 participants completed the trial without major protocol violations, forming the PP analysis group.

Two participants who only received an assigned number without receiving the investigational drug were excluded from the ITT analysis. During the clinical trial, a total of 6 participants were also excluded, including participants from the treatment group. The reasons for exclusion were the following: two participants violated the selection criteria based on estradiol levels; two participants were concurrently using prohibited medications; and two participants were excluded based on the researcher’s judgment.

A total of 52 participants were registered at Korea University Medical Center, with 52 participants from Inha University Hospital. The demographic and clinical history characteristics were analyzed in the ITT analysis population, which was the target for efficacy evaluation. The demographic characteristics are presented in Table 1. All participants in this clinical trial were female, and there were no statistically significant differences observed between the treatment group and the control group.

### 3.2. Primary Endpoint

The primary endpoint evaluation of this trial used the change in Kupperman index scores from baseline to the final evaluation at 12 days as the outcome variable. To demonstrate non-inferiority between the study group and the control group, the lower limit of the two-sided 95% confidence interval for the difference between groups was calculated. The results of the primary endpoint evaluation for the per-protocol (PP) analysis population, i.e., the main analysis population for efficacy evaluation, are shown in Table 2. The change in Kupperman index scores from baseline to 12 days after treatment in the PP analysis population, including 47 participants in the treatment group and 49 participants in the control group, showed a lower limit of the two-sided 95% confidence interval of −0.826. The lower limit of the one-sided 97.5% confidence interval for the difference in mean change in the Kupperman index between the two groups is −2.70. Since this value is greater than the non-inferiority margin of −3.2, it can be concluded that the experimental group demonstrates non-inferiority compared to the control group. The analysis of the ITT analysis population, which included 51 participants in the study group and 51 participants in the control group, also showed a lower limit of the two-sided 95% confidence interval for the change in Kupperman index scores from baseline to 12 days after treatment of 0.789. The lower limit of the one-sided 97.5% confidence interval for the difference in mean change in the Kupperman index between the two groups was −3.46 (Table 2).

### 3.3. Secondary Endpoints

Secondary endpoint evaluations were performed for the changes in E2 levels, FSH levels, total occurrences of daytime and nighttime flushes, and total scores of daytime and nighttime flushes from baseline to 12 days after treatment. The analysis did not show any statistically significant differences between the treatment group and the control group for all evaluation variables (Table 3). In the ITT group, the change in E2 levels 12 days after administering the test drug compared to baseline (screening) was −1.77 ± 8.60 pg/mL for the test group and −4.11 ± 26.46 pg/mL for the control group. There were no statistically significant changes within each group or significant differences between the groups (*p* = 0.587). The results for the PP group and the analysis divided by institution were similar to those of the ITT group. Furthermore, when analyzing the change in E2 levels with the test institution and premedication as covariates, both the ITT group (*p* = 0.589) and the PP group (*p* = 0.605) showed no significant differences between the groups. Regarding FSH levels, in the ITT group, the change in FSH levels 12 days after administering the test drug compared to baseline was 4.18 ± 14.59 mIU/mL for the test group and 2.45 ± 13.93 mIU/mL for the control group. There were no statistically significant changes within each group or significant differences between the groups (*p* = 0.561). The results for the PP group and the analysis divided by institution were similar to those of the ITT group. Additionally, when analyzing the change in FSH levels with the test institution and premedication as covariates, both the ITT group (*p* = 0.554) and the PP group (*p* = 0.607) showed no significant differences between the groups. The occurrence of daytime and nighttime flushes was analyzed based on the total number of occurrences on the day before the visit. In the ITT group, the change in the number of daytime flushes 12 days after administering the test drug compared to baseline (visit 2, day 0) was 2.81 ± 2.88 occurrences/day for the test group and 2.77 ± 2.91 occurrences/day for the control group. Both groups showed statistically significant changes within the group (*p* < 0.001), but there was no significant difference between the groups (*p* = 0.946). The results for the PP group and the analysis divided by institution were similar to those of the ITT group. Furthermore, when analyzing the change in the number of daytime flushes with the test institution and premedication as covariates, both the ITT group (*p* = 0.915) and the PP group (*p* = 0.842) showed no significant differences between the groups. In the ITT group, the change in the number of nighttime flushes 12 days after administering the test drug compared to baseline (visit 2, day 0) was 1.77 ± 3.05 occurrences/day for the test group and 1.54 ± 1.74 occurrences/day for the control group. Both the test group (*p* = 0.001) and the control group (*p* < 0.001) showed statistically significant changes within the group, but there was no significant difference between the groups (*p* = 0.637). The results for the PP group and the analysis divided by institution were similar to those of the ITT group. Additionally, when analyzing the change in the number of nighttime flushes with the test institution and premedication as covariates, both the ITT group (*p* = 0.8713) and the PP group (*p* = 0.696) showed no significant differences between the groups. In the ITT group, the change in total scores of daytime flushing after 12 days of administering the test drug compared to baseline (visit 2, day 0) was 5.81 ± 6.00 points for the test group and 5.71 ± 6.36 points for the control group. Both groups showed a statistically significant within-group change with *p* < 0.001, but there was no statistically significant difference between the groups (*p* = 0.937). The results for the PP group and the analysis divided by institution were similar to those of the ITT group. Furthermore, when analyzing the change in total scores of daytime flushing with test institutions and the presence of premedication as covariates, there was no statistically significant difference between the groups in both the ITT group (*p* = 0.981) and the PP group (*p* = 0.910). In the ITT group, the change in scores of nighttime flushing after 12 days of administering the test drug compared to baseline (visit 2, day 0) was 3.63 ± 6.55 points for the test group and 2.92 ± 3.59 points for the control group. Both the test group (*p* < 0.001) and the control group (*p* < 0.001) showed a statistically significant within-group change, but there was no statistically significant difference between the groups (*p* = 0.494). The results for the PP group and the analysis divided by institution were similar to those of the ITT group. Additionally, when analyzing the change in total scores of nighttime flushing with test institutions and the presence of premedication as covariates, there was no statistically significant difference between the groups in both the ITT group (*p* = 0.706) and the PP group (*p* = 0.529).

Therefore, when comparing Unicenta, the investigational drug, with the control drug Melsmon, it is demonstrated that Unicenta is non-inferior in terms of the menopausal symptom score (Kupperman index). Additionally, there were no statistically significant differences in hormone levels (estradiol, FSH) or in the evaluation of facial flushes.

### 3.4. Safety Analysis

Safety analysis, including adverse events, was conducted on a total of 102 participants in the safety group. During the clinical trial period, a total of 16 adverse events were reported. The incidence rate in the test group was 11.76% (6/51 individuals) with a frequency of eight events, while in the control group, it was 7.84% (4/51 individuals) with a frequency of eight events. There was no statistically significant difference in the incidence rate of adverse events between the two groups (*p* = 0.505). Regarding adverse drug reactions classified as “possibly related” to the investigational drug, the incidence rate in the test group was 1.96% (1/51 individuals) with a frequency of one event, while in the control group, it was 3.92% (2/51 individuals) with a frequency of two events. There was no statistically significant difference in the incidence rate of adverse drug reactions between the two groups (*p* = 0.558). No serious adverse events occurred during the clinical trial period.

## 4. Discussion

This clinical trial was a multicenter, randomized, double-blind, parallel-group, prospective study conducted at a total of two domestic institutions. The study aimed to evaluate the comparative efficacy and safety of Unicenta compared to Melsmon in women with menopausal symptoms. The study enrolled women with menopausal symptoms, and there were no statistically significant differences observed in the Kupperman index scores, which serve as indicators of menopausal symptoms, between the treatment group and the control group. Additionally, there were no differences observed between the groups in terms of demographic characteristics. A total of 104 participants agreed to participate in the trial and completed the screening period. Among them, 102 participants in the Safety analysis group received at least one dose of the investigational drug. Six participants in the Safety group did not meet the criteria specified in the trial protocol, resulting in a final count of 96 participants (PP set). The overall dropout rate of the clinical trial was 7.69%. There were no statistically significant differences observed among the participating hospitals in terms of the number of recruited participants, age, height, weight, past medical history, current medical history, or concomitant medication use. However, there was a statistically significant difference in terms of prior medication use. Additional analysis using prior medication use as a covariate did not reveal any statistically significant differences in efficacy evaluation variables between the two groups. The primary efficacy evaluation was based on the improvement in the Kupperman index. The change in the Kupperman index score from baseline to the final evaluation at 12 days after drug administration was analyzed. In the PP set, the mean Kupperman index change from baseline to day 12 showed statistically significant improvements. The lower limit of the one-sided 97.5% confidence interval for the difference in mean Kupperman index change between the two groups confirmed non-inferiority of the test group when compared to the control group. Similar results were also observed in the ITT analysis. Secondary efficacy endpoints included changes in E2 and FSH levels, frequency, and score of daytime and nighttime hot flashes. There were no statistically significant differences observed between the test and control groups in terms of E2 change or FSH change. However, both groups showed statistically significant improvements in the frequency of daytime hot flashes, frequency of nighttime hot flashes, total score of daytime hot flashes, and score of nighttime hot flashes. These results were consistent with the PP analysis and the stratified analysis by institution. No statistically significant differences were observed in the demographic baseline characteristics that showed statistical significance, including prior medication use, test group status, and trial institution, when analyzed using covariance analysis. From these results, it is evident that Melsmon is significantly effective in improving the symptoms of menopausal disorders. Furthermore, it leads to an overall improvement.

Menopausal disorders mainly occur due to abnormal secretion and dysfunction of the autonomic nervous system caused by a decline in ovarian function. Additionally, there are reports indicating that ovarian deficiency resulting from surgical castration shows differences in secretion status and biological response to external hormones. However, both cases exhibit similar symptoms and are therefore treated using the same methods. Placental extract developed by W.P. Filatov, the founder of corneal transplantation, has been treating menopausal disorders as an aging phenomenon through the activation of cellular respiration, reticulum endothelium system activation, and wound healing, based on “organizational therapy”. This approach differs from traditional medicine, which treats menopausal disorders by activating a wide range of biological processes. The endocrine function of the placenta is widely known. Trophoblast cells synthesize several hormones, such as estradiol, progesterone, and chorionic gonadotropin, which regulate fetal growth and development and maternal physiological changes during pregnancy [5]. Estradiol stimulates proliferation of the endometrium and mammary glands, promotes calcium retention, exhibits feminizing and anti-sclerotic effects, and influences sexual behavior. The main function of progesterone is to support the occurrence and maintenance of pregnancy. Exogenous administration of human chorionic gonadotropin stimulates ovulation, ovarian estrogen synthesis in females, and androgen synthesis and sperm production in males [4].

Views on the mechanism of action of placental preparations have been evolving [6]. Researchers have attempted to explain the therapeutic effects of placental derivatives based on the composition of vitamins, minerals, proteins, peptides, cytokines, and hormones. Such approaches aim to elucidate the common features of placental derivatives and shed light on their mechanism of action and unique biological activity.

Placental extracts have been primarily used in the treatment of various pathological conditions, most commonly in surgery, neurology, gynecology, and dermatology. Pronounced positive effects have been observed in the treatment of wounds, non-healing ulcers, and burns [7]. The rate of epithelialization significantly increases, and there is a decrease in inflammation and pain syndrome. The application of placental extracts in menopausal disorders has also proven beneficial [8]. It helps reduce the frequency and severity of hot flushes and irritability and normalizes hormonal profiles [9,10]. Experimental studies have shown an increase in estrogen receptors, a reduction in the effects of vaginal atrophy, and improved activity of osteoblasts. In a randomized controlled trial conducted on Korean women, menopausal symptoms and fatigue in middle-aged Korean women improved after 8 weeks of treatment. Risk factors for cardiovascular disease did not change during the study period [11] However, this study used a menopause rating scale to compare the efficacy. The reliability and validity of the MRS have not yet been verified in Korea. Another study investigated the effect of porcine placental extract on menopausal symptoms. Oral administration of porcine placental extract reduced menopausal symptoms in women with a BMI of 23 kg/m^2^ or higher or in early menopausal women [12]. The Kupperman index was used as a comparative tool for the evaluation of symptom changes. However, the efficacy of placental extract was only demonstrated for part of the participants. A recent study evaluated the effect of Hominis placenta extract pharmacopuncture for hot flashes in peri- and post-menopausal women [13]. After treatment, the hot flash score decreased significantly in both treatment and control groups (*p* < 0.001). However, there were no statistically significant differences in the mean changes in MRS, FSH, and E2 between the treatment and control groups. In this study, the Kupperman index showed improvement in both the Unicenta group and the Melsmon group in the overall study population evaluation. The results of the study provide evidence for the beneficial effects of placental extract on menopausal symptoms.

The use of the Kupperman index to assess menopausal symptoms was proposed by Kupperman in a study of American women [14]. The modified Kupperman index (mKMI) was developed to overcome the shortcomings of the original index, primarily the lack of assessment for vaginal dryness and loss of libido [15]. Therefore, we used the mKMI to evaluate menopausal symptoms in the current study with the intention of obtaining results that are different from those of previous studies.

There are several limitations in the current study. First, our study did not include other races. The evaluation of possible variation according to the race could be helpful. The second limitation of this study is a relatively short duration of the study. Further study with a longer duration and diverse race is warranted to confirm the efficacy of placental extract. Last, the third limitation is the need for frequent repetitive drug administration. The development of more concentrated and long-lasting medication could increase medication compliance and promote widespread use.

## 5. Conclusions

In conclusion, in this clinical trial involving women with menopausal symptoms, Unicenta was demonstrated to be non-inferior to Melsmon in terms of improvement in menopausal symptoms based on the evaluation of the change in the Kupperman index after six doses of the investigational drug administered for 12 days. There were no statistically significant differences observed in hormone levels or facial flushing between the two groups. Furthermore, there were no statistically significant differences in vitality signs or laboratory test items when comparing the two groups, indicating comparable safety. Therefore, Unicenta has shown effective improvement in menopausal disorder symptoms when compared to Melsmon.

## Figures and Tables

**Table 1:** Baseline characteristics of the participants in the intent-to-treat population.

	Unicenta (*n* = 51)	Melsmon (*n* = 51)	*p* Value
Age			
Mean, SD	55.10, 4.41	55.60, 5.66	0.617
Median (Min–Max)	54 (46–67)	56 (43–66)	
Weight (kg)			
Mean, SD	52.27, 10.00	58.31, 5.68	0.514
Median (Min–Max)	55.35 (44.0–96.8)	58.95 (46.5–71.0)	
Height (cm)			
Mean, SD	156.48, 4.42	155.6, 5.02	0.342
Median (Min–Max)	156.0 (143.0–166.0)	155.5 (142.0–166.0)	
History of illness (*n*, %)	27 (51.92%)	25 (48.08%)	0.695
Concurrent illness (*n*, %)	21 (40.38%)	29 (55.77%)	0.116
History of any drug (*n*, %)	21 (40.38%)	43 (65.38%)	0.011
Concurrent drug (*n*, %)	15 (28.85%)	22 (42.31%)	0.152

*n*: number, SD: standard deviation, Min: minimum, Max: maximum.

**Table 2:** Comparison of the change in the Kupperman index from baseline to visit 8 according to the intent-to-treat, per-protocol population.

Visit	Unicenta	Melsmon	*p* Value *
PP	(*n* = 47)	(*n* = 49)	
Baseline (mean, SD)	30.64, 6.79	31.31, 6.26	
Visit 8 (mean, SD)	13.38, 6.57	14.39, 5.33	
[Baseline—Visit 8] (mean, SD)	17.26, 7.64	16.92, 7.33	0.826
*p* value ^†^	<0.001	<0.001	
ITT	(*n* = 51)	(*n* = 51)	
Baseline (mean, SD)	30.83, 6.51	31.63, 6.30	
Visit 8 (mean, SD)	14.14, 7.29	14.59, 5.79	
[Baseline—Visit 8] (mean, SD)	16.63, 8.08	17.04, 7.42	0.789
*p* value ^†^	<0.001	<0.001	

* Students’ *t*-test, ^†^ Paired *t*-test [Baseline—visit8] gap between two groups = ([Baseline—visit 8] in Treatment group)—([Baseline—visit 8] in Control group). ITT: intent-to-treat, PP: per-protocol.

**Table 3:** Comparison of the secondary efficacy endpoint in the intent-to-treat population.

	Unicenta	Melsmon	*p* Value
E2 change (Mean, SD)	−1.77, 8.60	−4.11, 26.46	0.587
FSH change (Mean, SD)	4.18, 14.59	2.45, 13.93	0.561
Hot flash number change during daytime (Mean, SD)	2.81, 2.88	2.77, 2.91	0.946
Hot flash number change during night (Mean, SD)	1.77, 3.05	1.54, 1.74	0.637
Hot flash index change during daytime (Mean, SD)	5.81, 6.00	5.71, 6.36	0.937
Hot flash index change during night (Mean, SD)	3.63, 6.55	2.92, 3.59	0.494

## Data Availability

The data presented in this study are available upon request from the corresponding author.
